# Prevalence and Correlates of Sext-Sharing Among a Representative Sample of Youth in the Netherlands

**DOI:** 10.3389/fpsyg.2021.655796

**Published:** 2021-05-10

**Authors:** Sarah Boer, Özcan Erdem, Hanneke de Graaf, Hannelore Götz

**Affiliations:** ^1^Public Health Service Rotterdam-Rijnmond, Rotterdam, Netherlands; ^2^Rutgers, Utrecht, Netherlands

**Keywords:** sexting, non-consensual sharing, social media, adolescents, online sexual risk behavior

## Abstract

Many adolescents use their electronic devices to send each other sexually explicit texts, photos, and videos of themselves—commonly known as sexting. This can be fun and is not usually problematic. However, if the intended recipient decides to share these sexts with a broader audience, the consequences for the depicted can be detrimental. The purpose of this study was to investigate the prevalence of (non-consensual) sext-sharing among Dutch adolescents and explore the characteristics of those who do, to gain a better understanding of factors involved in dissemination. We used data from “Sex under the age of 25,” a representative national survey on sexual health among a sample of 20,834 Dutch 12–24-year-olds. The prevalence of sext-sharing was estimated using Complex Samples. Logistic regressions were used to assess associations between demographics, school-based sexting education, sexual- and online behavior, and mental health and sext-sharing. About 4% of the adolescents reported having shared someone else's sext in the last six months. Being male, aged 12–14 years, frequent social media usage, watching online porn, sexual experience, and being subjected to sext-sharing themselves associated most strongly with sext-sharing. Our findings show that the likelihood of sext-sharing is lower in older adolescents and that it associates with the extent of adolescents' sexual curiosity and online activity. The overlap between sharing sexts of others and having one's own sext shared suggests that dissemination of personal sexual content might be normalized or used as an act of retribution. Further research could be helpful to explain the mechanisms underlying this overlap. The results of this study illustrate the importance of exposing adolescents to evidence based preventive educational interventions on sexting from 12 years onwards and not just within the context of traditional school-based sex education, but also as a part of the (online) media-literacy curriculum.

## Introduction

On average U.S teens spend over 7 h on their screens per day (excluding homework), of which 70 min are spent on social media platforms. With a social life that increasingly takes place via the internet, it should come as no surprise that young people also use their electronic devices to explore their sexuality and send each other sexually explicit texts, photos, and videos of themselves—commonly referred to as sexting (Lenhart, 2009; Mitchell et al., 2012; Gámez-Guadix et al., 2017; Molla-Esparza et al., 2020). A meta-analysis of studies from 2009 to 2016 estimated that the lifetime prevalence of sexting among adolescents is about 15% for sending a sext (image, video, and/or message), and 27% for receiving one (Madigan et al., 2018). Findings from the most recent Dutch periodical sexual health survey “Sex under the age of 25” confirm that sexting has increased since 2012. Within the 6 months prior to the survey in 2017, 12% of the 12–24-year-olds had sent a sext (a nude image or sex-video) of themselves to someone else. This rate doubled since 2012 (De Graaf et al., 2017). Young people engage in sexting with platonic friends, casual flings and desired or established romantic partners to receive positive affirmation of their physical appearance, have fun, express affection, or elicit sexual desire (Anastassiou, 2017). Especially sexual minorities seem to rely on the internet for exploration of their sexuality and to meet romantic partners, as these prove more difficult for them to do in the physical domain (Hillier et al., 2012; Harper et al., 2016; Döring and Rohangis, 2019). Sexting is often labeled as a positive experience (Naezer, 2017).

Previously, the public discourse about sexting predominantly emphasized the associated risks and recommendations for abstinence. In recent years there has been increasing consensus on a normalcy perspective. In this perspective voluntary and consensually sharing sexually explicit imagery is seen as contributory to teenagers' sexual development and as a normal form of sexual self-expression and exploration (Döring, 2014; Lippman and Campbell, 2014; Naezer, 2017). The high lifetime prevalence of sexting among older age groups, suggesting it to be an accepted form of romantic interaction in adult life, further supports the perception that sexting plays a common role in the coming-of-age of sexually developing adolescents (Döring, 2014; Klettke et al., 2014; Döring and Rohangis, 2019). However, there is an agreement in the field that boundaries are crossed when consent is lacking and it is therefore essential to distinguish between consensual and non-consensual sexting (Albury and Crawford, 2012; Döring, 2014; Hasinoff, 2015).

Despite a bill to change this^1^ the Dutch laws that apply to sexting among minors do not yet discriminate between consensual sexting and non-consensual sexting. Officially, all forms of sexting are still punishable for this age group, because legally it is not yet distinguished from child pornography (article 240b sr, Penal Code). For adults, on January 1st 2020 a law was passed that makes sexting a punishable offense when it is done without consent or if the perpetrator could have known that sharing could be harmful for the person portrayed^2^^,^^3^.

Sharing a sext with others beyond the dyadic relationship exposes the depicted to intolerable risks. Once a sext gets into circulation, dissemination can go fast (Garcia et al., 2016; Van Ouytsel et al., 2017) and this has been known to result in reputational damage, self-blame, feelings of threat and paranoia, embarrassment and even suicide (Bates, 2016; Anastassiou, 2017). The Dutch “Sex under the age of 25” survey has demonstrated that having one's own sext shared with someone else than the intended recipient was generally disliked by the young people who experienced it (De Graaf et al., 2017). Despite the risks, sharing sexts with a wider audience is not uncommon. A meta-analysis on sexting behaviors among adolescents found a mean lifetime prevalence of 12% of forwarding a sext without consent (Madigan et al., 2018). This is not necessarily done with malicious intent. It is likely that adolescents do not always grasp the serious nature of sharing received sexts with others (Barrense-Dias et al., 2019; Clancy et al., 2019; Walker et al., 2019).

Since an effective way to avoid unwanted exposure is to prevent sexts from being shared, it is important to understand the characteristics of those sharing sexts and conditions facilitating this behavior (Walker and Sleath, 2017; Madigan et al., 2018; Naezer and Oosterhout Van, 2020).

Our review of the literature showed that until recently, primary sexting (sending and receiving) among adolescents has received the bulk of attention (Barrense-Dias et al., 2017) and that existing studies on sext-sharing often focus on the (usually female) victims exposed in non-consensually shared sexts (Livingstone and Smith, 2014; Branch et al., 2017; Clark et al., 2018). However, attention for perpetrators is growing. Existing research for instance suggests that non-consensual sharing is more common among men, non-heterosexuals (Barrense-Dias et al., 2019; Powell et al., 2019; Ruvalcaba and Eaton, 2020), older adolescents (Molla-Esparza et al., 2020) and by those with lower levels of education (Barrense-Dias et al., 2020). To our knowledge there is no information available about non-consensual sexting among ethnic minority groups in countries with a comparably diverse population as the Netherlands. We do know however, that Antillean adolescents, especially boys, are generally more involved in sending and receiving sexts than their peers (De Graaf et al., 2017).

Non-consensual sext-sharing can be considered as an act of bullying (Finkelhor et al., 2020). Ojeda et al. (2019) found a relationship between bullying and forwarding of sexts and point to the common characteristics between the two, such as a power imbalance and abuse thereof. Former studies on bullying found that the perpetrators more often have poorer mental health and that victims and perpetrators are not seldomly the same person (Kowalski and Limber, 2013). There are indications that these associations also apply to non-consensual sext-sharing. Barrense-Dias et al. (2019) indeed found a link between forwarding sexts and poorer mental health and the overlap between victimization and perpetration in the unwanted distribution of sexually explicit material is revealed in multiple recent studies (Powell et al., 2019; Walker et al., 2019; Clancy et al., 2021).

Several studies describe an association between (non-consensual) sexting and other forms of sexual activity, although the link with porn consumption is inconsistent (Clancy et al., 2019; Raine et al., 2020). However, Van Oosten and Vandenbosch (2020) demonstrated that porn consumption and a higher instrumental attitude (i.e., seeing sex as purely physical, fun and exciting) toward sex, can increase willingness to forward sexts of strangers. There is some evidence to suggest that online porn consumption is related to viewing women as sex objects (Brown and L'Engle, 2009; Flood, 2009; Peter and Valkenburg, 2009), which could potentially lower inhibitions to share nude images with others. Vanden Abeele et al. (2014) draw attention to the similarities in group dynamics of online porn consumption and sexting, in which both behaviors coexist with a greater need for popularity and both are used as a currency to gain social status within the peer group.

Sexting is a social affair that often takes place through social network applications like Snapchat, WhatsApp, and Facebook. The available literature supports the notion that a higher involvement in sexting goes hand in hand with a higher smartphone usage and increased social engagement in general (Baumgartner et al., 2014; Yépez-Tito et al., 2020). More specifically, associations were established between sexting and a higher internet use, excessive texting and a more frequent use of WhatsApp and Snapchat (Rice et al., 2014; West et al., 2014; Yépez-Tito et al., 2020) and between forwarding sexts and more social network applications used (Molla-Esparza et al., 2020).

Lastly, our literature review demonstrated that both in the Netherlands and internationally, there is a wide range of school-based interventions aimed at preventing online sexual violence, including unwanted sext sharing. Many of these programs center around sexting abstinence and the use of fear appeals about the risks, to discourage sexting altogether (Oosterwijk and Fischer, 2017; Finkelhor et al., 2020). Experts question the effectiveness of programs that promote abstinence, and according to them, methods that are evidence-based are currently lacking (Döring, 2014; Finkelhor et al., 2020). It is further argued that strongly promoting abstinence from sexting as the sole solution to prevent dissemination might contribute to victim-blaming. It could involuntarily convey the notion that it is foolish to exchange sexts and therefore someone's own fault when their sext is shared with a broader audience (Setty, 2019). The only study that we could find that examined the relationship between sext-sharing and sexting prevention education, found that those who had received lessons about sext-sharing, were slightly more likely to have shared sexts. This might suggest that sexting is sometimes discussed in response to an incident (Johnson et al., 2018).

The purpose of this study was to investigate the prevalence of (non-consensual) sext-sharing among Dutch adolescents and explore the characteristics of those who do, to gain a better understanding of factors involved in dissemination. We included known correlates of (non-consensual) sexting from earlier studies and looked at the relationship between sext-sharing and socio-demographic characteristics (gender, age, ethnic background, urbanity, and sexual orientation); school based education about sexting; online behavior (social media usage, dating apps); sexual activity (watching online porn, experience with partner sex); victimization of non-consensual sexting (unwanted exposure to sexts, having your own sext shared) and mental health. For this we used data from the Dutch national sexual health survey “Sex under the age of 25–2017”.

Our findings will be useful to identify directions for follow-up research and ultimately contribute to the development of evidence-based interventions to prevent sext-sharing.

Given earlier findings as discussed above, we expected that adolescents who are male, older, have a gay-bisexual orientation, show more online activity, are more sexually active, have been victims of non-consensual sexting themselves and have poorer mental health are more likely to share-sexts. Despite the existing doubts about the effectiveness of present educational programs on sexting, the basic assumption remains that these programs should reduce the risks of sexting. Therefore, we also expected that having received education about sexting would show a lower likelihood of sharing sexts.

## Materials and Methods

### Ethics Statement

The study proposal was submitted to The Medical Research Ethics Committee of the University Medical Center Utrecht and exempt from ethical review (reference number Wag/mb16/013562).

### Study Population

Data for this study were collected as part of “Sex under the age of 25,” a large representative national sexual health study in 2017 among youth aged 12–24 years in the Netherlands (De Graaf et al., 2017).

#### Sampling Procedure

Participants were recruited by means of high schools and the Dutch population register. Respondents aged 12–16 years were recruited from the first four grades of selected high schools. To create a representative sample that reflected the geographical and educational distribution of young people in the Netherlands, we stratified all Dutch secondary schools by geographic region and educational level. From those strata we invited a random selection of schools to participate. For every school that refused to participate a substitute school was recruited from the same stratum.

The sample of 17–24-year-olds was drawn from the population register by Statistics Netherlands. We approached this age group with a postal letter, and two reminders, inviting them to fill out an online questionnaire. To account for the anticipated lower response rate among adolescents with a non-Western background, this group was oversampled (Ahlmark et al., 2015). Incentives varied between Public Health Service regions, but the majority of the selected young people received a voucher worth €5, which they could cash irrespective of participation.

#### Final Study Sample

From the 361 schools we approached, 106 participated in the study (response rate: 29%). In total, 4,927 adolescents aged 12–16 years filled out our questionnaire. Of the 92,399 17–24-year-olds that were invited to fill out the online survey, 17,227 (18.6%) responded.

About 6% of the respondents (*n* = 1,320) were excluded from the original sample because they indicated that they did not answer all questions honestly or because there were more than two inconsistencies in their responses. Respondents were also excluded if a parent had filled in the questionnaire or if they did not speak Dutch. The remaining analytical sample consisted of 20,834 respondents; comprising 4,846 respondents of 12–16 years old and 15,988 respondents of 17–24 years old. To adjust for selective non-response and overrepresentation of certain regions, the data were weighted for geographical location, gender, age and educational level. Further details about recruitment and the sample can be found elsewhere (De Graaf et al., 2017, 2018).

Of the 20,834 potential respondents, no data were missing on the outcome measure. The percentage of missing data of the independent variables were all below 5% (Table 1) and ranged from 0% for most socio-demographic variables to 3.3% for watching online porn. In the multivariate analyses, participants with missing data on one or more variables were excluded.

**Table 1:** Sample characteristics of 20,834 adolescents in the Netherlands in 2017 and the weighted prevalence of sext-sharing.

	**Total sample**		**Shared sext** **<** **6 months**	
**Variable**	**n**^**a**^	**Weighted**^**b**^**(%)**	**Weighted** ^**b**^**(%)**	**95% CI**
Total	20,834	100	4.2	3.7–4.7
**Sex (0% missing)**				
Female	12,653	49.4	2.1	1.7–2.6
Male	8,181	50.6	6.3	5.4–7.2
**Age group (0% missing)**				
12–14 years	2,980	22.9	3.5	2.6–4.5
15–17 years	4,154	23.1	5.7	4.7–6.9
18–20 years	6,268	22.1	4.1	3.3–5.1
21–24 years	7,432	31.8	3.7	2.9–4.8
**Education (8% missing)**				
Less	7,926	55.7	5.0	4.2–5.8
More	12,740	44.3	3.2	2.7–3.7
**Ethnic background (0% missing)**				
Dutch/Western background	18,517	85.0	4.0	3.5–4.5
Turkish	326	2.4	1.8	0.7–4.6
Moroccan	265	2.1	7.1	3.3–14.6
Surinamese	472	3.0	3.0	1.7–5.1
Antillean	227	1.2	11.8	6.3–20.9
Other non-Western	1027	6.4	6.1	3.7–9.8
**Urbanity (6% missing)**				
Urban	10,785	48.3	3.7	3.1–4.5
Urban-rural	3,383	18.7	4.3	3.4–5.5
Rural	6,533	33.0	4.4	3.6–5.4
**Sexual orientation (2.3% missing)**				
Heterosexual	19,496	96.1	4.1	3.6–4.6
Gay/Bisexual	854	3.9	8.2	4.7–13.8
**School-based sexting education (0% missing)**				
Sufficient	5,313	28.9	5.3	4.4–6.4
Non or little	15,521	71.1	3.8	3.2–4.4
**Time spent on social media (0.5% missing)**				
< 1 h p/d	2,602	13.6	2.4	1.3–4.4
1–3 h p/d	9,882	45.4	3.5	2.9–4.2
3 or more hours p/d	8,253	41.0	5.6	4.9–6.5
**Ever used a dating app (0% missing)**				
No	12,900	66.9	3.4	2.9–3.9
Yes	7,933	33.1	5.9	5.0–7.0
**Watching online porn-videos** **<** **6 months (3.3% missing)**				
Never	8,506	43.2	1.9	1.4–2.4
Up to once p/w	8,550	40.0	4.9	4.2–5.8
Multiple times p/w	3,090	16.9	9.2	7.5–11.3
**Experience with partner sex (0% missing)**				
None	6,138	38.2	2.0	1.5–2.6
Little	2,019	11.0	6.8	5.2–8.9
Advanced	12,677	50.8	5.3	4.6–6.2
**Unwanted exposure to sexts** **<** **6 months (0% missing)**				
No	19,659	94.3	4.0	3.5–4.5
Yes	1,174	5.7	8.0	5.7–11.1
**Own sext shared by others** **<** **6 months (0% missing)**				
No	20,056	95.4	3.5	3.1–4.0
Yes	777	4.6	19.0	14.9–23.9
**Mental health (in tertiles) (1.8% missing)**				
High	5,716	31.6	4.0	3.3–5.0
Middle	7,917	36.6	3.2	2.6–3.9
Low	6,819	31.7	5.4	4.5–6.6

a *Number of participants do not always add up to total N due to missing values*.

b *Calculated using SPSS complex samples, weighted for geographical location, gender, age and educational level*.

### Measures

The questionnaire commenced with several sociodemographic characteristics such as gender, age, educational level, and ethnic background followed by a broad range of topics like relational involvement, sexual experiences, online behavior and mental health, with tailored questions according to previous answers. We will now further describe the variables we used in this study, starting with the outcome measure.

*Sext-sharing*. The outcome variable was measured by asking whether the respondent had ever sent a nude image or sex-video of someone else within the last six months (1 = *never*, 2 = *once*, 3 = *more than once*). The answers were then dichotomized (0 = *never* and 1 = *once/more than once*).

*Sexual orientation*. To assess sexual orientation respondents were asked: “Do you feel attracted to boys, girls or both?” (1 = *only boys*, 2 = *mainly boys, but also girls*, 3=*equally to boys and girls*, 4 = *mainly girls, but also boys*, 5 = *only girls, 6* = *none, 7* = *undecided*). The outcomes were then dichotomized accordingly for boys and girls into (1 = *only or mainly attracted to the opposite sex* 2 = *equally, mainly or only attracted to the same sex*).

*School-based sexting education*. Respondents were asked to rate the amount of information they had received in school about several subjects. One of these subjects was:” Sending nude images or sex-videos” (1 = *none*, 2 = *little*, 3 = *sufficient*). The outcomes were then dichotomized (1 = *sufficient* and 2 = *none/little*).

*Time spent on social media*. The quantity of time spent on social media was determined by asking respondents: “On average, how much time do you spend on social media each day?” (1 = *less than 1 hour*, 2 = *1–3 h*, 3 = *3–5 h*, 4 = *5–10 h*, 5 = *10 h or more*). The outcomes were then trichotomized (1 = *less than 1 h*, 2 = *1–3 h*, 3 = *3 or more hours*).

*Using dating apps*. Dating app usage was measured by letting respondents indicate which of the listed dating apps (*Tinder, Happn, Grindr, Badoo, Hot or not, Inner Circle; another dating app, namely…*) they had ever used. If they had never used a dating app, respondents could check the option:” I have never used a dating app.” The outcomes were then dichotomized (1 = *never used a dating app* and 2 = *used one or more dating apps*).

*Watching online porn*. Online porn consumption was measured by letting respondents indicate how often within the last 6 months they had viewed online porn-videos (1 = *never*, 2 = *less than once a month*, 3 = *1–3 times a month*, 4 = *once a week*, 5 = *multiple times a week*). The outcomes were trichotomized (1 = *never watched online porn-videos*, 2 = *watched online porn videos up to once a week*, 3 = *watched online porn videos multiple times per week*).

*Experience with partner sex*. According to respondents' answers to dichotomized questions about several sexual experiences (1 = *no*, 2 = *yes*), respondents were divided into three levels of experience [1 = *none* (kissing, masturbation), 2 = *some* (petting, manual sex), 3 = *advanced* (oral sex, intercourse, anal sex)].

*Unwanted exposure to sexts*. A composite variable was created to determine whether a respondent had been exposed to sexually explicit images, videos or video-chats and disliked it. For this variable we used a combination of variables measuring whether within the last 6 months the respondent had 1. received personal nude images or sex-videos or 2 saw someone's genitals during a video-chat or 3 saw someone masturbate during a video-chat (1 = *never*, 2 = *once*, 3 = *more than once*) and, if applicable, variables measuring how the respondent had experienced this (1 = *I liked it*, 2 = *I did not like/dislike it* and 3 = *I disliked it*). Based on the answers to these questions, respondents were divided into two categories (1 = *not exposed to unwanted sexts* and 2 = *exposed to unwanted sexts*).

*Having one's own sext shared*. To identify respondents who experienced having their own sext shared, a composite variable was created. This variable combined two variables measuring whether within the last 6 months the respondent had experienced that someone had 1. showed his/her sext to someone else or 2. forwarded his/her sext to someone else (1 = *never*, 2 = *once*, 3 =*more than once)*. The variable was then dichotomized (1 = *never* and 2 = *once/more than once)*.

*Mental health*. We included the Kessler Psychological Distress Scale as an overall measure for mental health; its properties are described elsewhere (Kessler et al., 2002; Fassaert et al., 2009). Because one item was accidentally omitted, we used the overall mean score of the 9 remaining items (0–5) which were then divided into tertiles (1 = *high*, 2 = *middle* and 3 = *low*).

### Statistical Analyses

We used the Complex Samples module to generate the weighted prevalence of recent sext-sharing by subgroups (Table 1). Further, we performed multivariable logistic regression analyses for which we used unweighted data but included variables that were used for the weighting factor as independent variables in our models (Table 2). This is the preferred approach when sample weights are not a function of the dependent variable in the model (Winship and Radbill, 1994). To mitigate the impact of influential observations on our regression model we used bootstrapping, a technique that uses random resampling to produce regression estimates that are more resistant to outliers in the data (Efron and Tibshirani, 1994). A correlation matrix of the independent variables can be found in Supplementary Material A. All our analyses were performed in IBM SPSS statistics 27.

**Table 2:** Multivariate associations with sext-sharing within the last 6 months (95% percentile bootstrap confidence intervals based on 1,000 samples).

	**Model 1 (*****N*** **=** **20,533)**			**Model 2 (*****N*** **=** **19,003)**		
**Variable**	**aOR**	**95% CI**	***p***	**aOR**	**95% CI**	***p***
**Sex**						
Female	1			1		
Male	**2.69**	2.27–3.20	0.001	**2.19**	1.76–2.72	0.001
**Age group**						
12–14 years	1			1		
15–17 years	**1.89**	1.45–2.51	0.001	0.78	0.55–1.13	0.165
18–20 years	1.33	1.01–1.81	0.050	**0.40**	0.28–0.60	0.001
21–24 years	1.11	0.84–1.48	0.491	**0.37**	0.25–0.56	0.001
**Education**						
Less	1			1		
More	**0.69**	0.58–0.80	0.001	**0.82**	0.70–0.98	0.023
**Ethnic background**						
Dutch/Western background	1			1		
Turkish	0.46	0.10–0.92	0.057	0.45	0.10–1.01	0.088
Moroccan	1.39	0.66–2.30	0.275	**2.01**	0.91–3.52	0.027
Surinamese	**1.63**	0.96–2.46	0.036	1.43	0.83–2.24	0.139
Antillean	**2.30**	1.13–3.70	0.002	**2.11**	0.97–3.70	0.016
Other non-Western	**1.41**	0.99–1.92	0.039	1.38	0.90–1.93	0.083
**Urbanity**						
Urban	1			1		
Urban-rural	1.24	0.98–1.53	0.059	**1.43**	1.13–1.79	0.007
Rural	1.13	0.92–1.35	0.226	**1.31**	1.07–1.58	0.010
**Sexual orientation**						
Heterosexual				1		
Gay/bisexual				1.21	0.81–1.66	0,269
**School-based sexting education**						
Sufficient				1		
Non or little				0.88	0.74–1.08	0.195
**Time spent on social media**						
< 1 h p/d				1		
1–3 h p/d				**1.65**	1.14–2.51	0.010
3 or more hours p/d				**2.87**	2.04–4.42	0.001
**Ever used a dating app**						
No				1		
Yes				**1.44**	1.20–1.74	0.001
**Watching online porn-videos** **<** **6 months**						
Never				1		
Up to once p/w				**1.66**	1.32–2.16	0.001
Multiple times p/w				**2.68**	2.00–3.67	0.001
**Experience with partner sex**						
None				1		
Little				**2.70**	1.86–3.90	0.001
Advanced				**3.64**	2.67–5.13	0.001
**Unwanted exposure to sexts** **<** **6 months**						
No				1		
Yes				**1.59**	1.17–2.11	0.002
**Own sext shared by others** **<** **6 months**						
No				1		
Yes				**4.31**	3.32–5.55	0.001
**Mental health (tertiles)**						
High				1		
Middle				1.08	0.86–1.35	0.483
Low				**1.31**	1.07–1.67	0.015

*Bold values indicate significant at p < 0.05 level*.

## Results

Table 1 presents the characteristics of the weighted sample and the weighted prevalence of recent sext-sharing by subgroup. Of the 12–24-year-olds, 4.2% had shared nude images or sex videos of someone else within the last six months. Sext-sharing was most prevalent among males (6.3%), youth with an Antillean background (11.8%), gay- and bisexuals (8.2%), those watching online porn multiple times a week (9.2%) and those who had been exposed to unwanted sexts (8.0%). However, the proportion was highest in the group whose own personal sext was shared by others recently (19.0%).

Table 2 shows the logistic regression results. Model 1 describes the strength of the associations between demographic variables and sext-sharing. It demonstrates that young males were almost three times more likely to share someone else's sext than their female counterparts (aOR = 2.69; 95% CI: 2.27–3.20). The likelihood of sext-sharing was higher among 15–17-year-olds, compared to the 12–14-year-olds (aOR = 1.89; 95% CI: 1.45–2.51), somewhat lower among more educated young people (aOR = 0.69; 95% CI: 0.58–0.80), compared to less educated participants and (somewhat) higher among young people with an other non-Western background (aOR = 1.41; 95% CI: 0.99–1.92), a Surinamese background (aOR = 1.63; 95% CI: 0.96–2.46), and an Antillean background (aOR = 2.30; 95% CI: 1.13–3.70), compared to those with a Dutch/Western background.

In model 2 we added the independent variables: sexual orientation, school-based sexting education, online behavior (social media usage, dating apps) sexual activity (watching online porn, experience with partner sex), victimization of non-consensual sexting (unwanted exposure to sexts, having your own sext shared) and mental health. Adding these variables to the model resulted in a decreased association with being male (aOR = 2.19; 95% CI: 1.76–2.72) and a shift to the youngest age group (12–14-year-olds) that was most likely to share a sext. Adolescents with a Moroccan and Antillean background showed to be twice as likely to share a sext as those with a Dutch/Western background. Model 2 further demonstrates that youth living in urban-rural and rural areas were more likely to share someone else's sext compared to their urban counterparts. We also found a strong association with porn consumption. Compared to those not watching online porn videos, the ones who did multiple times a week had a 2.68 higher likelihood of sext-sharing (95% CI: 2.00–3.67). With respect to other online behavior, model 2 shows that those who spent 3 or more hours a day on social media, had an adjusted odds ratio of 2.87 (95% CI:2.04–4.42) compared to those who spent less than one hour a day on social media. Most strongly associated with sext-sharing was the recent experience of having one's own sext shared by others (aOR = 4.31; 95% CI: 3.32–5.55), followed by advanced experience with partner sex compared to having no experience with partner sex (aOR = 3.64; 95% CI: 2.67–5.13). Sexual orientation and school-based sexting education were not significantly associated with sext-sharing.

## Discussion

The purpose of this study was to investigate the prevalence and correlates of sext-sharing among a nationally representative sample of youth living in the Netherlands. The results show that sext-sharing is not uncommon among Dutch youth, since 4.2% of the 12–24-year-olds shared a nude image or sex-video of someone else in the previous six months. The lifetime prevalence of sext-sharing is probably multiple times higher, as was found in a recent meta-analysis (Madigan et al., 2018). We found that males, 12–14-year-olds (when adjusted for other variables), less educated youth and adolescents with a Moroccan or Surinamese background are more likely to share someone else's sext. Furthermore, the use of social media, dating apps, watching online porn, having more experience with partner sex, unwanted exposure to sexts, having one's own sext shared and a lower mental health are all associated with a higher risk of sext-sharing. This is in line with our expectations. Contrary to what we anticipated, we did not find an association between sexual orientation or having received school-based sexting education and the likelihood of sext-sharing.

Males are twice as likely to share sexts than females. Earlier studies found that females were commonly judged more harshly whether they sexted (e.g., “slut”) or not (e.g., “prude”) compared to males, which could explain their reluctancy to share sexts of others (Lippman and Campbell, 2014). Males are also found to perceive sext-sharing more often as a common activity, whereas females feel that sexting is private behavior (Walker and Sleath, 2017). Furthermore, males sometimes engage in sext-sharing to brag about their capacities to obtain these images (Walker and Sleath, 2017), to strengthen the social bonds with their peer-group or increase social status (Bindesbøl Holm Johansen et al., 2018; Barrense-Dias et al., 2019; Clancy et al., 2019). This finding could also be explained by higher levels of sensation-seeking and lower levels of impulse control which are both more prevalent in males than in females (Shulman et al., 2015).

Whereas the prevalence of general forms of sexting is higher in older age groups (De Graaf et al., 2017), when sexting increasingly occurs within the context of a romantic relationship (Lippman and Campbell, 2014), our findings show that the risk of *sharing* is highest among the 15–17-year-olds. However, after controlling for other factors (e.g., online behavior and sexual activity), the highest risk shifts from age 15 to 17 to age 12–14. Apparently, the primary higher prevalence of sext-sharing among 15–17-year-olds (partly) parallels their increased (sexualized) media usage or offline sexual activity. Given equal levels of other behaviors such as online and sexual activity among 12–14-year-olds, their risk of sext-sharing would be much higher. Young adolescents are especially vulnerable because their risk for harmful side-effects of sexting is higher, yet their risk perception is lower (Garcia et al., 2016; Barrense-Dias et al., 2017). This could also be explained from a neuropsychological perspective, which states that young adolescents make more impulsive decisions because of a maturational imbalance between the part of the brain that is sensitive for (short-term) rewards and the part involved in goal-directed behavior (Steinberg et al., 2008).

Young people's lives are embedded in their socio-cultural environment, which shapes their perspectives on sexuality. Cense (2019) argues that existing messages about sexuality in our western culture are often ethnocentric in nature and do not consider other realities with different values and morals with regards to sexuality. Second generation Moroccan youth often grow up in an environment where premarital sex is strongly disapproved of, especially for girls (Hendrickx et al., 2002). As a result, they are unlikely to adopt an (imposed) western point of view that acknowledges sexting as an accepted form of sexual self-expression between unmarried people. Disapproval of sexting in general, may lower reservations to share sexts that are already in circulation. The observation that Antillean adolescents are more likely to share sexts, may be explained by Antillean boys having a relatively large number of sex partners (De Graaf et al., 2017). It is important to reach young people with messages that match their socio-cultural realities. This calls for additional qualitative exploration, into the motives for sex-sharing, in which special attention is required for the perceptions, norms and experiences of young people from different cultural backgrounds.

We found that school-based sexting education was not significantly associated with sext-sharing. We cannot draw any conclusions about the effectiveness of existing school-based education based on this outcome, since we did not measure what the content of these lessons was. We also do not know if a formal educational program was used, or if only casual discussions were held. If a formal educational program was used, it is possible that these lessons focused on abstinence from sexting as a means to avoid risks, which is unlikely to prevent (non-consensual) sext sharing (Döring, 2014; Oosterwijk and Fischer, 2017; Finkelhor et al., 2020; Patchin and Hinduja, 2020). It is also conceivable that programs were used that were effective, but that the effects were neutralized by schools that only addressed the subject following an incident with sext-sharing (Johnson et al., 2018). Finally, we did not assess whether the topic was discussed one time or repeatedly. It is unlikely to find an effect of a single time intervention, since behavioral change requires a systematically developed intervention which is repeated at diverse moments in the school-career (Finkelhor et al., 2020).

Our findings do confirm an association between sext-sharing and other forms of sexual activity. The link with frequent porn consumption could originate from the fact that both sexting and watching porn are signs of an increasing interest in sexuality. Pornography is primarily used to stimulate sexual arousal (Sun et al., 2016). It is conceivable that sexts are sometimes exchanged among friends to elicit sexual arousal. A normalization of non-consensual sharing or dissemination of sexts could also be suggestive of some sort of sexual objectification or an instrumental attitude (Van Oosten and Vandenbosch, 2020). Further research could illuminate if there are shared attitudes that can explain the connection between porn consumption and sext-sharing and if so, how these are related to both behaviors. For example, a high instrumental attitude could simultaneously precede porn consumption and sext-sharing, but it could also be a mediating factor.

The connection we found with experience with partner sex parallels the findings in earlier studies that sexting and offline sexual behaviors coincide (Baumgartner et al., 2012; Raine et al., 2020). Possibly, both sext-sharing and offline sexual behavior are forms of sexual experimentation and a reflection of a developmentally normative interest in sexuality. One study found that the likelihood of getting sexually active was higher for young people who had previously sent a nude picture of themselves. This suggests that sexting could be a form of preparatory behavior or a sign that someone is ready to have offline sex. Another explanation can be found in the routine activity theory, which assumes that a person is more likely to offend if he or she has more opportunities to do so (Pratt and Turanovic, 2016). More sexually experienced young people could have more opportunities for sext-sharing than inexperienced young people. This theory could also underpin the correlation between time spent on social media and sext-sharing.

Of all factors included in the present study, the recent experience of having one's own sext shared associates most strongly with sharing someone else's sexts. One theory that could explain this association is the general strain theory (Agnew, 1992). This theory states that the negative treatment by others generates negative emotions, which in turn instigate negative actions. If sexts are exchanged within a romantic relationship, and one of the partners betrays the other one's trust, it is conceivable that the latter retaliates. Retribution could also play a role in the association we found between sext-sharing and unwanted exposure to sexts. Naezer and Oosterhout Van (2020) describe that some of their respondents forwarded unsolicited sexts because they felt harassed by the sender and wanted to teach him a lesson.

### Strengths and Limitations

One of the strengths of our study was the use of a representative national sample which enabled us to gain insight in the prevalence of sext-sharing among Dutch youth. However, despite using sampling weights to increase the representativeness of the sample, it is conceivable the sample still differs from the study population on characteristics that could not be weighed for. Also, only a minority of the schools that were approached to take part in this study, participated. This has possibly resulted in a selective sample of schools, as schools with a more permissive school climate toward sexuality or an increased emphasis on sexual health in their curriculum may have been more likely to participate.

Secondly, the nature of our outcome measure (i.e., “Have you ever sent nude pictures or videos of someone else”) has a few limitations. It is possible that the prevalence we found is an underestimation, if some of the respondents felt inclined to give a socially desirable answer despite the anonymity they were guaranteed. Additionally, the formulation of the question is not entirely unambiguous, since the question does not include consent. In theory this question could also be answered affirmative if someone took a consensual photo or video of someone else and then shared the result only with the person depicted in the sext. Furthermore, we cannot rule out that “someone else” is in some cases someone depicted on a publicly available image, for example leaked celebrity sex-tapes. Since the outcome variable was part of a series of questions relating to sharing personal sexually explicit content, we however believe this specific interpretation of the question is unlikely. A strength but also a weakness is the narrow definition of the outcome measure. We excluded sexual text messages from the outcome measure because explicit images or videos are believed to have a potentially greater impact than sharing sexual text messages (Houck et al., 2014). This is in agreement with other studies about non-consensual sext-sharing (Walker and Sleath, 2017; Clark et al., 2018; Johnson et al., 2018). A consequence of this is that it limits comparability of our results with studies that do include sexual text messages in their outcome measure.

In our study we followed the dichotomous gender classification (male/female) that was customary at the time. Unfortunately, as a result it remains unclear to what extent non-binary, transgender and intersex youth are involved in sext-sharing and this should be addressed in future research.

Another limitation is that our dataset is cross-sectional, making any conclusions about causality impossible. To illustrate this, in the association found between the variable *mental health* and sext-sharing, sext-sharing can be the cause, outcome or both, or both variables can be related to a confounding variable. Since this research was explorative in nature, no comprehensive framework could be applied to this study which can be considered to be a limitation as well.

Finally, the original Kessler psychological distress scale (which was used to assess mental health) consists of 10 items. One item (i.e., “During the past 30 days, how often did you feel nervous?”) was accidentally omitted from the questionnaire. Fortunately, Cronbach's Alpha of the remaining 9 items was still high (0.92).

In conclusion, our findings warrant additional research to improve the understanding of the mechanisms underlying the victim-offender overlap between those sharing sexts and those having their sext shared. Additional research could possibly also shed more light on the reasons for not finding a positive relationship between school-based sexting education and the sharing of sexts. The results further illustrate the importance of targeting adolescents from a young age (from 12 years onwards) with educational interventions specifically addressing the risks of sharing someone else's sext. This is important because sext-sharing by young people often seems to result from an underestimation of the possible consequences rather than from bad intentions (Clancy et al., 2019). Targeting adolescents at a young age will also increase the probability of being able to address the subject preventively rather than as a response to unfavorable incidents. In the Netherlands ensuring social safety is an obligatory part of the responsibilities of schools^4^. This includes the protection of adolescents against (non-consensual) sext-sharing and other types of (cyber)bullying, requiring effective school-based educational interventions. In these interventions, a shift in focus is recommended from the initial senders to the recipients and factors that facilitate further dissemination. It is also advised to concentrate on teaching young people skills to minimize the harms that may result from sexting, rather than to advocate abstinence. Furthermore, the importance is emphasized of addressing the normative aspects of sexting and to convey the notion that a sext is private and that it is never OK to share received sexts with someone else (Finkelhor et al., 2020; Naezer and Oosterhout Van, 2020; Patchin and Hinduja, 2020). Our findings suggest that sext-sharing closely links to sexual activity as well as with other online social and romantic ventures. It also shares characteristics with other forms of bullying. It is therefore recommendable to not include the subject of (non-consensual) sext-sharing in school-based sexual health education exclusively, but to also integrate the topic in media-literacy lessons teaching adolescents how to use (online) media safely, responsibly and respectfully as well as in traditional school based anti-bullying programs. This way, the subject can receive the repeated attention that is required for effectiveness. For the development of evidence and theory-based interventions we suggest the application of the Intervention Mapping Approach (Bartholomew Eldredge et al., 2016; Mevissen et al., 2018). This approach uses a thorough, systematic and detailed protocol to guide intervention development, implementation and evaluation and has been widely used to plan health promotion programs in the Netherlands.

## Data Availability Statement

All partners involved in this study (Rutgers, SoaAids Nederland and the Public Health Services) share ownership of the data used for his study. Permission from them would be needed before sharing this dataset with third parties. Any data-requests would also need to comply with existing data-sharing regulations as stipulated by the municipality of Rotterdam. Requests to access the dataset should be directed to Sarah Boer, se.boer@rotterdam.nl.

## Ethics Statement

The study proposal was submitted to The Medical Research Ethics Committee of the University Medical Center Utrecht and exempt from ethical review (reference number Wag/mb16/013562). Written informed consent from the participants' legal guardian/next of kin was not required to participate in this study in accordance with the national legislation and the institutional requirements.

## Author Contributions

SB and ÖE initiated the study, all authors discussed the design. SB, ÖE, and HdG collaborated in the data-preparation and analysis. Results were interpreted by all authors. SB wrote the introduction, methods, and result section and HdG wrote the discussion of the manuscript. HG provided expert input and supervised the collaboration process. All authors contributed to drafting and revision of the paper and all authors approved the final manuscript.

## Conflict of Interest

The authors declare that the research was conducted in the absence of any commercial or financial relationships that could be construed as a potential conflict of interest.

## Footnotes

^1^https://www.rijksoverheid.nl/actueel/nieuws/2020/05/12/grapperhaus-moderniseert-wetgeving-seksueel-grensoverschrijdend-gedrag.

^2^https://www.rijksoverheid.nl/onderwerpen/seksuele-misdrijven/wraakporno.

^3^https://www.government.nl/latest/news/2019/09/24/grapperhaus-several-offences-to-receive-higher-maximum-sentence.

^4^https://www.rijksoverheid.nl/onderwerpen/veilig-leren-en-werken-in-het-onderwijs/veiligheid-op-school.
